# Supplementary material for: Presence and Multi-Species Spatial Distribution of Oropouche Virus in Brazil within the One Health Framework
Source: Trop Med Infect Dis. 2022 Jun 20;7(6):111. doi: 10.3390/tropicalmed7060111 (PMC9230142; doi:10.3390/tropicalmed7060111)
Supplement: Supplementary file 1 [file tropicalmed-07-00111-s001.zip › tropicalmed-1728374-supplementary.pdf]

Tropical Medicine and Infectious Disease

MDPI

Systematic Review

Presence and Multi-Species Spatial Distribution of Oropouche Virus in Brazil within the One Health Framework

Sofia Sciancalepore [1,2,*], Maria Cristina Schneider [1,3], Jisoo Kim [2], Deise I. Galan [1] and Ana Riviere-Cinnamond [2]

1 Department of International Health, Georgetown University, Washington, DC 20057 USA; mcs368@georgetown.edu (M.C.S.); dgl32@georgetown.edu (D.I.G.)
2 Health Emergency Department, Pan American Health Organization (PAHO/WHO), Washington, DC 20037 USA; kimjis@paho.org (J.K.); rivierea@paho.org (A.R.-C.)
3 Institute of Collective Health Studies, Federal University of Rio de Janeiro 21941-901, Brazil
* Correspondence: sofiasciancalepore@gmail.com or ss3510@georgetown.edu

Citation: Sciancalepore, S.; Schneider, M.C.; Kim, J.; Galan, D.I.; Riviere-Cinnamond, A. Presence and Multi-Species Spatial Distribution of Oropouche Virus in Brazil within the One Health Framework. *Trop. Med. Infect. Dis.* **2022**, *7*, 111. https://doi.org/10.3390/tropicalmed7060111

Academic Editors: Frank Badu Osei and Santanu Sasidharan

Received: 29 April 2022
Accepted: 17 June 2022
Published: 20 June 2022

Publisher's Note: MDPI stays neutral with regard to jurisdictional claims in published maps and institutional affiliations.

Copyright: © 2022 by the authors. Licensee MDPI, Basel, Switzerland. This article is an open access article distributed under the terms and conditions of the Creative Commons Attribution (CC BY) license (https://creativecommons.org/licenses/by/4.0/).

Table S1. PRISMA checklist [13].

Section and Topic	Item #	Checklist item	Location where item is reported
**TITLE**			
Title	1	Identify the report as a systematic review.	Lines 1-2
**ABSTRACT**			
Abstract	2	See the PRISMA 2020 for Abstracts checklist.	Lines 9-22
**INTRODUCTION**			
Rationale	3	Describe the rationale for the review in the context of existing knowledge.	Lines 44-52, 57, 62- 64
Objectives	4	Provide an explicit statement of the objective(s) or question(s) the review addresses.	Line 91-96
**METHODS**			
Eligibility criteria	5	Specify the inclusion and exclusion criteria for the review and how studies were grouped for the syntheses.	Lines 111-124
Information sources	6	Specify all databases, registers, websites, organisations, reference lists and other sources searched or consulted to identify studies. Specify the date when each source was last searched or consulted.	Lines 106-110
Search strategy	7	Present the full search strategies for all databases, registers and websites, including any filters and limits used.	Lines 108-109, 111-112
Selection process	8	Specify the methods used to decide whether a study met the inclusion criteria of the review, including how many reviewers screened each record and each report retrieved, whether they worked independently, and if applicable, details of automation tools used in the process.	Lines 111-121
Data collection process	9	Specify the methods used to collect data from reports, including how many reviewers collected data from each report, whether they worked independently, any processes for obtaining or confirming data from study investigators, and if applicable, details of automation tools used in the process.	Lines 128-137

Section and Topic	Item #	Checklist item	Location where item is reported
Data items	10a	List and define all outcomes for which data were sought. Specify whether all results that were compatible with each outcome domain in each study were sought (e.g. for all measures, time points, analyses), and if not, the methods used to decide which results to collect.	Supplementary materials
	10b	List and define all other variables for which data were sought (e.g. participant and intervention characteristics, funding sources). Describe any assumptions made about any missing or unclear information.	Supplementary materials
Study risk of bias assessment	11	Specify the methods used to assess risk of bias in the included studies, including details of the tool(s) used, how many reviewers assessed each study and whether they worked independently, and if applicable, details of automation tools used in the process.	Supplementary materials
Effect measures	12	Specify for each outcome the effect measure(s) (e.g. risk ratio, mean difference) used in the synthesis or presentation of results.	Not applicable
Synthesis methods	13a	Describe the processes used to decide which studies were eligible for each synthesis (e.g. tabulating the study intervention characteristics and comparing against the planned groups for each synthesis (item #5)).	Supplementary materials
	13b	Describe any methods required to prepare the data for presentation or synthesis, such as handling of missing summary statistics, or data conversions.	Lines 128-137
	13c	Describe any methods used to tabulate or visually display results of individual studies and syntheses.	Lines 138-150
	13d	Describe any methods used to synthesize results and provide a rationale for the choice(s). If meta-analysis was performed, describe the model(s), method(s) to identify the presence and extent of statistical heterogeneity, and software package(s) used.	Lines 138-142
	13e	Describe any methods used to explore possible causes of heterogeneity among study results (e.g. subgroup analysis, meta-regression).	Not applicable
	13f	Describe any sensitivity analyses conducted to assess robustness of the synthesized results.	Not applicable
Reporting bias assessment	14	Describe any methods used to assess risk of bias due to missing results in a synthesis (arising from reporting biases).	
Certainty assessment	15	Describe any methods used to assess certainty (or confidence) in the body of evidence for an outcome.	Supplementary material

Section and Topic	Item #	Checklist item	Location where item is reported
**RESULTS**			
Study selection	16a	Describe the results of the search and selection process, from the number of records identified in the search to the number of studies included in the review, ideally using a flow diagram.	Lines 152-174
	16b	Cite studies that might appear to meet the inclusion criteria, but which were excluded, and explain why they were excluded.	Supplementary materials
Study character-istics	17	Cite each included study and present its characteristics.	Supplementary materials
Risk of bias in studies	18	Present assessments of risk of bias for each included study.	Not applicable
Results of indi-vidual studies	19	For all outcomes, present, for each study: (a) summary statistics for each group (where appropriate) and (b) an effect estimates and its precision (e.g. confidence/credible interval), ideally using structured tables or plots.	Lines 193-200, 202- 210, 218-229
Results of syn-theses	20a	For each synthesis, briefly summarise the characteristics and risk of bias among contributing studies.	
	20b	Present results of all statistical syntheses conducted. If meta-analysis was done, present for each the summary estimate and its precision (e.g. confidence/credible interval) and measures of statistical heterogeneity. If comparing groups, describe the direction of the effect.	Not applicable
	20c	Present results of all investigations of possible causes of heterogeneity among study results.	Not applicable
	20d	Present results of all sensitivity analyses conducted to assess the robustness of the synthesized results.	Not applicable
Reporting biases	21	Present assessments of risk of bias due to missing results (arising from reporting biases) for each synthesis assessed.	
Certainty of evi-dence	22	Present assessments of certainty (or confidence) in the body of evidence for each outcome assessed.	Supplementary materials
**DISCUSSION**			

Section and Topic	Item #	Checklist item	Location where item is reported
Discussion	23a	Provide a general interpretation of the results in the context of other evidence.	Lines 326-375
	23b	Discuss any limitations of the evidence included in the review.	Lines 392-398
	23c	Discuss any limitations of the review processes used.	Lines 379-390
	23d	Discuss implications of the results for practice, policy, and future research.	Lines 403-417
**OTHER INFORMATION**			
Registration and protocol	24a	Provide registration information for the review, including register name and registration number, or state that the review was not registered.	Not applicable
	24b	Indicate where the review protocol can be accessed, or state that a protocol was not prepared.	Not applicable
	24c	Describe and explain any amendments to information provided at registration or in the protocol.	Not applicable
Support	25	Describe sources of financial or non-financial support for the review, and the role of the funders or sponsors in the review.	Line 425
Competing interests	26	Declare any competing interests of review authors.	Lines 435-437
Availability of data, code and other materials	27	Report which of the following are publicly available and where they can be found: template data collection forms; data extracted from included studies; data used for all analyses; analytic code; any other materials used in the review.	Supplementary materials

Table S2. Documented presence of Oropouche virus by species by state, Brazil, 1960- 2018.

		Acre	Amapa	Amazonas	Bahia	Goias	Maranhao	Mato Grosso	Mato Grosso do Sul	Minas Gerais	Para	Rondonia	Tocantins	Rio Grande do Sol	Sao Paulo
**Human**	*Homo sapiens*	1	1	1	1	1	1	1	0	0	1	1	1	0	1
**Non-human Primates**	*Alouatta caraya* (Black howler monkey)	0	0	0	0	1	0	0	0	0	0	0	0	1	0
	Alouatta guariba clamitans (Southern brown howler monkey)	0	0	0	0	0	0	0	0	0	0	0	0	1	0
	Callithrix penicillata (Black-tufted marmoset)	0	0	0	0	0	0	0	0	1	0	0	0	0	0
	Cebus libidinosus (Black- striped capuchin)	0	0	0	0	1	0	0	0	0	0	0	0	0	0

	Cebus apella (Tufted capuchin)	0	0	0	0	1	0	0	1	0	0	0	0	0	0
	Sapajus apella (Robust tufted capuchin)	0	0	0	0	0	0	0	1	0	0	0	0	0	0
	Callithrix sp. (Monkey)	0	0	0	0	0	0	0	0	1	0	0	0	0	0
**Midge & Mosquito**	*Aedes aegypti* (Mosquito)	0	0	0	0	0	0	1	0	0	0	0	0	0	0
	Culex quinquefasciatus (Mosquito)	0	0	0	0	0	0	1	0	0	1	0	0	0	0
	Culicoides paraensis (Midge)	0	0	0	0	0	1	0	0	0	1	0	0	0	0
	Ochlerotatus serratus (Mosquito)	0	0	0	0	0	0	0	0	0	1	0	0	0	0
**Sloth**	*Bradypus tridactylus*	0	0	0	0	0	0	0	0	0	1	0	0	0	0
**Not identified**	N/A	1	1	1	1	0	1	1	0	0	1	1	1	0	0
**Other**	*Columbina talpacoti* (Bird/ dove species)	0	0	0	0	0	0	0	0	0	1	0	0	0	0

Fringillidae, Thaurapidae (Bird/ finch species)	0	0	0	0	0	0	0	0	0	1	0	0	0	0
Unknown wild bird species	0	0	0	0	0	0	0	0	0	0	0	0	0	0
Unknown domestic bird species	0	0	0	0	0	0	0	0	0	1	0	0	0	0
Proechimys sp. (Rodent/ rat species)	0	0	0	0	0	0	0	0	0	1	0	0	0	0
Sheep	0	0	0	0	0	0	0	1	0	0	0	0	0	0
**References**	a	b	c	d	e	f	g	h	i	j	k	l	m	n

[a] References for the State of Acre [1,3,5,24,26,32,33,35,38,41]
[b] References for the State of Amapa [1,22,26,28,32]
[c] References for the State of Amazonas [1,3–5,18,20,24–25,26,29,31–32,35,36,38,41,43,45,50,52]
[d] References for the State of Bahia [1,21]
[e] References for the State of Goias [1,42,44,52]
[f] References for the State of Maranhao [3,5,24,26,32,35,38,41]
[g] References for the State of Mato Grosso [1,4,27,39]
[h] References for the State of Mato Grosso do Sul [1,30,40,46]
[i] References for the State of Minas Gerais [1,3,5,24,26,28,32,35,50]
[j] References for the State of Para [1,3–5,19,24,26,32,34–35,38,41,45,52,54]
[k] References for the State of Rondonia [1,3–5,24,26,32,35,38,41]
[l] References for the State of Tocantins [1,5,26,32–33,35]
[m] References for the State of Rio Grande do Sul [47]
[n] References for the State of Sao Paulo [4]

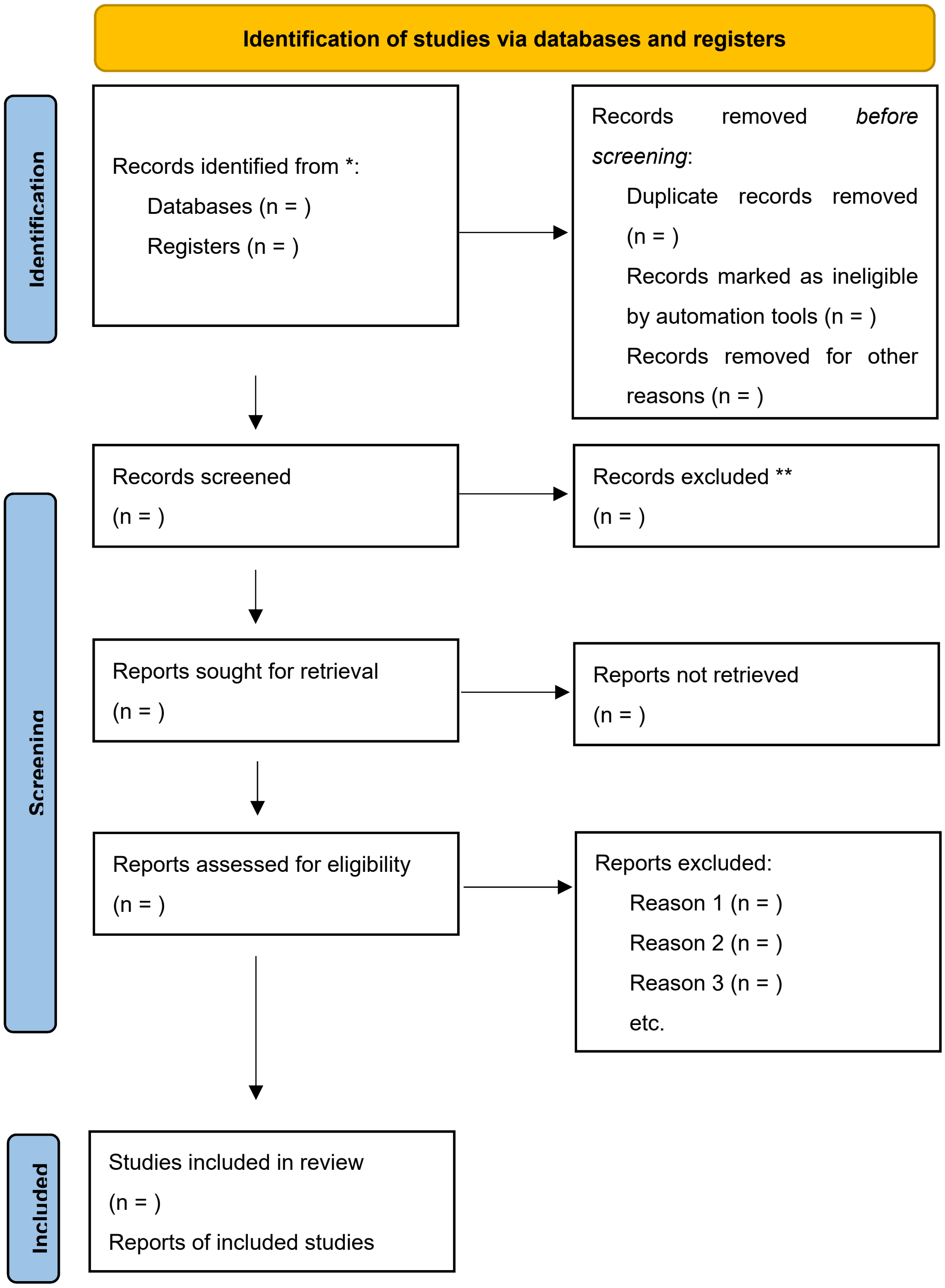

Figure S1. PRISMA flow chart diagram template [13]. * Consider, if feasible to do so, reporting the number of records identified from each database or register searched (rather than the total number across all databases/registers). ** If automation tools were used, indicate how many records were excluded by a human and how many were excluded by automation tools.

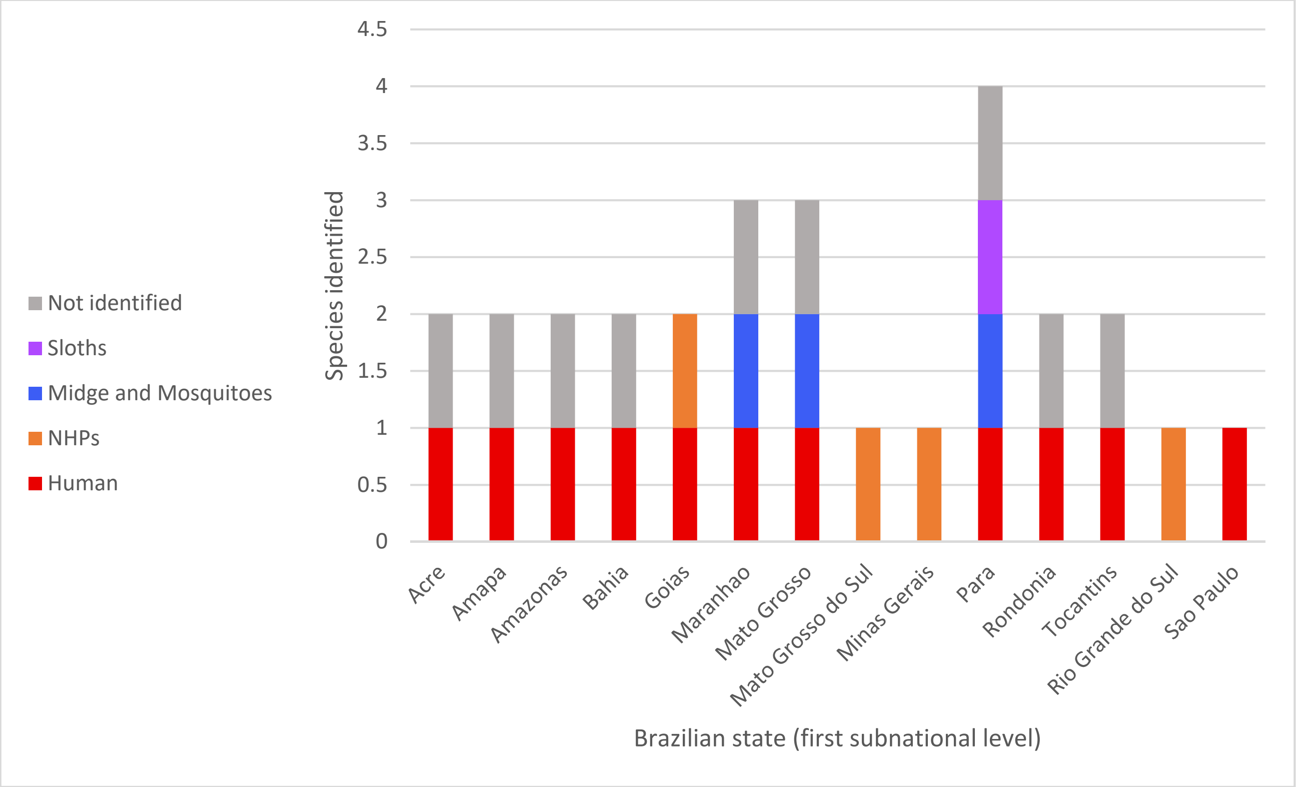

Figure S2. Species of detection of Oropouche virus by state, Brazil, 1960- 2018 [1,3–5,18–54].
